# Functional Tea Extract Inhibits Cell Growth, Induces Apoptosis, and Causes G0/G1 Arrest in Human Hepatocellular Carcinoma Cell Line Possibly through Reduction in Telomerase Activity

**DOI:** 10.3390/foods13121867

**Published:** 2024-06-14

**Authors:** Yuan Chen, Changsong Chen, Jiaxing Xiang, Ruizhen Gao, Guojun Wang, Wenquan Yu

**Affiliations:** 1 Tea Research Institute, Fujian Academy of Agricultural Sciences, Fuzhou 350003, China; chenyuan@faas.cn (Y.C.); 19949533447@163.com (J.X.); 18639424217@163.com (R.G.); 2 Fujian Academy of Agricultural Sciences, Fuzhou 350003, China; 3 Agricultural Product Processing Research Institute, Fujian Academy of Agricultural Sciences, Fuzhou 350003, China; 4 Horticulture College, Fujian Agriculture and Forestry University, Fuzhou 350003, China; 5 Harbor Branch Oceanographic Institute, Florida Atlantic University, 5600 U.S. 1, Fort Pierce, FL 34946, USA; guojunwang@fau.edu

**Keywords:** hepatocellular carcinoma, Hep3B, growth inhibition, apoptosis, cell cycle arrest, telomerase

## Abstract

The functional tea CFT-1 has been introduced into China as a nutraceutical beverage according to the “Healthy China” national project. The effects on human hepatocellular carcinoma (HCC) cells remain unclear and were investigated with the functional tea extract (purity > 98%). The morphological changes in the cells were observed with microscopes. Cell proliferation, migration, cycle distribution, and apoptotic effects were assessed by MTT, Transwell assays, and flow cytometry, respectively, while telomerase inhibition was evaluated with telomerase PCR ELISA assay kits. The CFT-1 treatment resulted in cell shrinkage, nuclear pyknosis, and chromatin condensation. CFT-1 suppressed the growth of Hep3B cells with IC50 of 143 μg/mL by inducing apoptosis and G0/G1 arrest in Hep3B cells. As for the molecular mechanism, CFT-1 treatment can effectively reduce the telomerase activity. The functional tea extract inhibits cell growth in human HCC by inducing apoptosis and G0/G1 arrest, possibly through a reduction in telomerase activity. These results indicate that CFT-1 extract exhibited in vitro anticancer activities and provided insights into the future development and utilization of CFT-1 as functional foods to inhibit the proliferation of HCC cells.

## 1. Introduction

Liver cancer is a frequently occurring malignancy with a poor prognosis and a high mortality rate, and is the second leading cause of cancer-related deaths [1]. Hepatocellular carcinoma (HCC), as an aggressive tumor, accounts for 90% of primary liver cancers and constitutes the third leading cause of cancer mortality worldwide [2,3,4]. The main therapeutic methods, including surgery, radiotherapy, and chemotherapy, often have limited effects on the tumors, but huge side effects on the human body. HCC is notoriously characterized by a poor prognosis and high mortality rate owing to the high rates of metastasis and recurrence [5,6,7]. There are a variety of risk factors, such as infection with viral hepatitis, hepatic cirrhosis, obesity, and consumption of dietary hepatocarcinogens [8,9]. According to epidemiologic studies, it has been shown that regular tea consumption is associated with a decreased risk of various carcinomas, including breast cancer [10], bladder or kidney cancer [11,12], liver cancer [13,14], lung cancer [15], upper aerodigestive tract cancer [16], and others. Therefore, cancer prevention is of great significance. One potential way to fight cancers is to develop a functional food that has a highly inhibitive effect on cancer cells but low toxicity on healthy cells.

As the most abundant and active ingredient in tea, (−)-epigallocatechin-3-gallate (EGCG) has raised considerable interest in the development of anti-cancer drugs [17,18,19]. Mechanism studies suggested that in a variety of cancer cells, EGCG inhibits telomerase activity by down-regulating the protein expression, which eventually leads to the suppression of cell viability and induction of apoptosis [20]. Telomerase, a ribonucleoprotein acting to elongate telomeres, has been directly implicated in tumorigenesis and shown to be expressed in approximately 90% of all cancers [21]. Accumulating literature has demonstrated the ability of EGCG to inhibit the growth and proliferation of hepatocellular tumors through the induction of apoptosis and modulation of autophagic and anti-angiogenetic activities [22]. Recently, a new tea plant strain, *Camellia sinensis CV,* was developed.CFT-1 extract (CFT-1) was introduced into China as a nutraceutical beverage according to the “Healthy China” national project, which aims at exploring the resources of crops that are rich in nutrients and functional ingredients. CFT-1 is rich in EGCG, which is almost twice as abundant as in the nationwide popular tea Fuyun No. 6 [23]. Furthermore, CFT-1 has larger amounts of antioxidants, which are believed to have benefits in terms of preventing cancer. However, to the best of our knowledge, there is no study exploring the health benefits or pharmacological activity of CFT-1, especially in the prevention and treatment of human HCC.

The aim of this study was to conduct a preliminary study on the antitumor activity and mechanism of CFT-1 extracts to support the development and synthesis of new, efficient, and low-toxicity anti-tumor lead compounds. We hypothesized that possible molecular mechanisms would be related to the effect of CFT-1 on telomerase activity.

## 2. Materials and Methods

### 2.1. Reagents

The experimental samples were extracted from the functional tea CFT-1 with purity >98%. Human Hep3B hepatoma cells were provided by the Fujian Medical University biochemistry and molecular biology laboratory. MTT (3-[4,5-dimethylthiazol-2-yl]-2,5-diphenyltetrazolium bromide) and all other chemicals employed in this study were of analytical grade and purchased from Sigma-Aldrich Co. Fetal bovine serum, Dulbecco’s Modified Eagle’s medium (DMEM), and penicillin–streptomycin were obtained from Thermo Fisher Scientific, Inc. Telomerase PCR ELISA kit was purchased from Boehringer Mannheim.

Gallic acid (GA), (−)-gallocatechin (GC), caffeine (CAF), theophylline (THEO), (−)-epigallocatechin (EGC), (+)-catechin (C), chlorogenic acid (CHL), theobromine (TB), caffeic acid (CAA), (−)-epicatechin (EC), (−)-epigallocatechin gallate (EGCG), ρ-coumaric acid (COU), (−)-gallocatechin gallate (GCG), ferulic acid (FER), sinapic acid (SIN), epicatechin gallate (ECG), rutin (RUT), myricetin (MYR), quercetin (QUE), and kaempferol (KAE) were purchased from Sigma (St. Louis, MO, USA), and the purity of the reagents was above 95%. The acetonitrile (HPLC grade) was purchased from Merck KgaA (Darmstadt, Germany), and all other reagents, including methanol and formic acid, were purchased from Sinopharm Chemical Reagent Co., Ltd. (Shanghai, China). Ultrapure water was obtained from a Milli-Q water system (Millipore, Bedford, MA, USA).

### 2.2. Preparation of CFT-1 Extract

Tea samples underwent initial drying at 35 °C for 2 h, followed by crushing into powders and passage through a 40-mesh screen (304 stainless steel sieve, Yongkang Jielong Industrial and Trade Co., Ltd., Jinhua, China). The selection of this specific mesh screen aimed to optimize the extraction procedure, achieving elevated dissolution rates while minimizing material loss. An aliquot of 3 g of sample powder was weighed into an Erlenmeyer flask, and 150 mL of water was added. The mixture was shaken for 15 min at room temperature and centrifuged at 8000 rpm for 15 min at 4 °C. This extraction process was repeated once. The supernatants from the two extracts were combined, diluted to 50 mL with water, and analyzed.

### 2.3. HPLC-DAD Analysis

In this study, a HPLC-DAD system was employed for the analysis. The instrument used for this analysis was the Ultimate 3000 HPLC (Thermo Fisher Scientific, Milan, Italy). Prior to HPLC analysis, the extract was filtered through a 0.22 µm microporous membrane, and 1 µL of the filtered extract was injected into the HPLC system. Chromato-graphic separation was performed using a reverse-phase column (Merck Lichrospher RP-18, 250 mm × 4.6 mm, Darmstadt, Germany). Mobile phases A and B were 0.1% formic acid and acetonitrile, respectively. The gradient elution procedure was: 0 min, 94% A; 11 min, 93% A; 12 min, 92% A; 18 min, 90% A; 48 min, 82% A; 56.8 min, 82% A; 58 min, 69% A; 68 min, 52% A; 70 min, 6% A. The analysis duration for each specimen was 20 min, inclusive of a 4 min column equilibration period. The column temperature and flow rate were maintained at 30 °C and 0.8 mL·min^−1^, respectively. For detection, two wavelengths, 280 and 340 nm, were compared in this study using the DAD integrated into the HPLC system. The developed HPLC method was validated according to ICH guidelines to ensure fulfillment of current regulatory standards.

### 2.4. Cell Culture and Treatment

Human HCC Hep3B cells were purchased from Shanghai Cell Bank, Chinese Academy of Sciences (Shanghai, China). Hep3B cells were cultured with DMEM supplemented with 10% heat-inactivated FBS and 1% penicillin–streptomycin solution in 5% CO_2_ at 37 °C. Human HCC cell line Hep3B was selected as our study model. To evaluate the effect of CFT-1 exact in HCC cell line Hep3B, the extract of functional tea CFT-1 (brown powder) was weighted and dissolved in a variety of volumes of Milli Q water to produce the desired drug concentrations (containing approximately 143 mg/g of EGCG). These samples were then ultrafiltered for the following use. For the CFT-1 treatment, the solution of CFT-1 extract was added to the Hep3B cells and incubated for 48 h followed by examinations. Cell morphology was examined and photographed using a Nikon TS2 inverted microscope at a magnification of ×100.

### 2.5. Cell Viability Assay

Hep3B cell viability was measured with an MTT assay according to previous publications. Briefly, Hep3B cells were seeded in 96-well culture plates at a density of 1 × 10^4^ cells per well. After incubation for 24 h, cells were treated with different concentrations of CFT-1 extract for 48 h. Then, 20 μL MTT solution (5 mg/mL) was added to each well, and the cells were incubated at 37 °C for an additional 4 h. After removing the supernatants, 200 μL dimethyl sulfoxide (DMSO) was added to each well. The optical density (OD) was measured at 450 nm using a microplate reader. Cell viability was calculated using the following formula:

Cell viability (%) = (OD_experimental group_ − OD_blank control_)/(OD_control group_ − OD_blank control_) × 100%

### 2.6. Colony Formation Assay

A colony formation assay was carried out according to a previous publication with minor modifications [24]. Specifically, 200 cells/10 mL of Hep3B were plated onto a 10 cm dish and incubated for 24 h prior to drug treatment. Then, the cells were exposed to CFT-1 extract followed by incubation for an additional 20 days. The colony formation ability was observed after fixing with 4% paraformaldehyde and staining with 0.1% crystal violet.

### 2.7. Transwell Migration Assay

The effect of CFT-1 extract on Hep3B cells was evaluated using a modified Boyden chamber model as previously reported [25]. The Transwell insert was placed back onto the 24-well plate, and the lower chamber was filled with 0.6 mL of DMEM containing 20% FBS with or without 143 μg/mL (IC50) of CFT-1. Human Hep3B cells (5 × 10^4^ cells/well) in 200 µL medium were plated to the upper chamber. After 5 h of incubation at 37 °C, all non-migrated cells were removed from the upper face of the Transwell membrane, and migrated cells were fixed with methanol, stained with 0.1% crystal violet, and counted under an Olympus light microscope at a magnification of ×100. The formula was calculated as: relative cell counts per field = number of migrated cells in sample/number of migrated cells in control × 100%.

### 2.8. Cell Cycle Analysis

The effect of CFT-1 extract on the cell cycle distribution was measured with PI staining according to a previous publication. Hep3B cells were treated with 143 μg/mL CFT-1 or vehicle control in 6-well culture plates for 48 h. Approximately 1 × 10^6^ cells were collected, washed with PBS, and fixed with 70% ethanol at −20 °C overnight. After centrifugation, cells were then resuspended in 1 mL staining solution (50 µg/mL PI, 20 µg/mL RNAase). After incubation for 30 min at room temperature in the dark, samples were analyzed with a flow cytometer. The percentage of cells in various phases of the cell cycle was determined using FlowJo software.

### 2.9. Apoptosis Analysis

Cell apoptosis was determined using an annexin V-FITC kit according to the manual. Briefly, Hep3B cells were treated with or without 143 μg/mL CFT-1 extract for 48 h prior to analysis. Cells were then collected and washed twice with cold PBS. Then, they were stained with Annexin V and PI in binding buffer at room temperature for 15 min. Cells were then analyzed using flow cytometry.

### 2.10. Telomerase Activity Assay

Telomerase activity in Hep3B cells was measured using a telomerase PCR ELISA assay kit according to the manual. Briefly, after treatment with CFT-1 extract or vehicle control for 48 h, the cells were collected, lysed, and homogenized in 200 μL of lysis buffer. After 30 min of incubation on ice, the lysates were centrifuged and the supernatants were collected. Protein concentration was measured using a BCA reagent. Then, 5 μg of cell extract was used in the following PCR amplification step, and aliquots (5 µL) of PCR product were analyzed using ELISA assay.

### 2.11. Statistical Analysis

All experiments were performed in triplicate, and the results are expressed as the mean ± SD. Data were analyzed using SPSS software. Comparisons between groups were performed using *t*-test. Differences were considered statistically significant at *p*-values < 0.05.

## 3. Results

### 3.1. Characterization of CFT-1 Tea Extract

Figure S1 shows the quantitative results of the identification of the compounds contained in CFT-1 extracts using HPLC. The concentrations of various compounds in the extract were measured and reported as follows (Table 1).

### 3.2. CFT-1 Treatment Induced Morphologic Alterations in Hep3B Cells

Morphology and structure are the material basis of function, and malignant tumor cells have typical cytological characteristics: a large nucleocytoplasmic ratio and multiple large nucleoli with nucleolar edge aggregation phenomenon, all of which are signs of rapid growth of tumor cells. The cell surface has dense and slender microvilli, which facilitate the exchange of substances between the tumor and the outside world for malignant proliferation, as well as facilitating metastasis and attachment to other tissues. Morphological differentiation and maturation are markers of malignant tumor cell differentiation.

**Figure 1:**
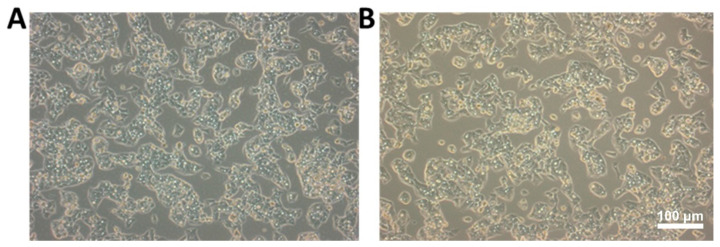
Morphology of cells treated with CFT-1 extract. (**A**) Hep3B cells treated with vehicle control. (**B**) Hep3B cells treated with CFT-1 extract (100 μg/mL). Scale bar = 100 μm.

We firstly investigated the effect of CFT-1 extract on Hep3B cells by examining the morphological changes in cells which were exposed to different concentrations of CFT-1. As shown in Figure 1, in comparison with the untreated cells (control group), Hep3B cells treated with CFT-1 extract (100 μg/mL) clearly showed enhanced amounts of shrinkage of the cell body, compaction of the nucleus, and condensation of chromatin, which together are regarded as typical apoptotic features [26]. On this basis, we were interested in investigating the anti-cancer activity of CFT-1 on Hep3B cells.

### 3.3. CFT-1 Treatment Suppressed the Cell Growth, Colony Formation, and Migration of Hep3B Cells

Suppression of cell growth, colony formation, and migration in Hep3B cells is a common goal in cancer research, aiming to develop strategies to inhibit the aggressive behavior of HCC, which is often associated with high proliferation and metastatic potential. Research on the effects of natural compounds on Hep3B cells often focuses on identifying substances with anti-cancer properties that can suppress cell growth, colony formation, and migration. EGCG has been investigated for its potential anticancer properties, including its ability to suppress cell growth and induce apoptosis in Hep3B cells.

Liver cancer cells grow actively, and the magnitude of absorbance values in MTT experiments can reflect the number of live cells. Therefore, the MTT colorimetric method can be used to quantitatively determine the effect of anticancer drugs on human cancer cells based on their quantity and metabolic activity [27]. We incubated Hep3B cells with CFT-1 at different concentrations (10, 30, 50, 70, 90, 100 μg/mL) for 48 h, and then examined the cell viability with MTT assay. It was seen that CFT-1 treatment obviously reduced the cell viability in a dose-dependent manner (Table 2). The half-maximal inhibitory concentration (IC50) of CFT-1 against Hep3B cells was about 143 μg/mL after 48 h incubation. It was noted that this potency is comparable to the reported activity of chemical EGCG on HCC cell lines (e.g., HepG2: IC50 = 74.7 µg/mL, SMMC7721: IC50 = 59.6 µg/mL; SK-hep1: IC50 = 61.3 µg/mL). As referenced above, EGCG is the major component of green tea, with great promise as a cancer preventive. Our results suggest that CFT-1 has great anti-cancer potential.

**Figure 2:**
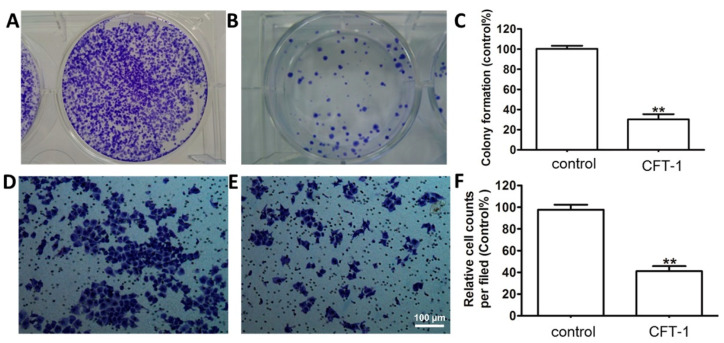
Effect of CFT-1 treatment on colony formation ability and mobility of Hep3B cells. (**A**–**C**) Colony formation of Hep3B cells treated with 143 μg/mL CFT-1. (**D**–**F**) Transwell assays demonstrating the migration ability of Hep3B cells treated by 143 μg/mL CFT-1. ** *p* < 0.01. Scale bar = 100 μm.

We also assessed the effect of CFT-1 on the colony formation of Hep3B cells. As shown in Figure 2, it can be seen the CFT-1 treatment (143 μg/mL, 48 h) dramatically limited the colony formation by reducing both the number and size of the colonies. In other words, CFT-1 treatment resulted in much fewer and smaller colonies compared to the untreated Hep3B cells (Figure 2A,B). Quantitative analysis indicated that the colony formation of Hep3B cells was reduced to 32.01% by CFT-1 (Figure 2C). Furthermore, CFT-1-treated cells appeared to be less densely packed as compared with untreated cells.

We further determined the influence of CFT-1 on the cell mobility with the Transwell migration assay. As shown in Figure 2D,E, the migration of Hep3B cells was significantly suppressed by CFT-1 treatment. Specifically, the number of migrated Hep3B cells in the treated group was reduced by more than 41.7% as compared to the vehicle control cells (Figure 2F).

### 3.4. CFT-1 Treatment-Induced Apoptosis and G0/G1 Arrest in Hep3B Cells

Apoptosis and G0/G1 arrest are two important cellular processes that researchers often study in the context of cancer, including in Hep3B cells, which are a commonly used HCC (liver cancer) cell line. These processes are relevant because they play key roles in controlling cell growth, preventing uncontrolled proliferation, and influencing the response to various treatments. Apoptosis, or programmed cell death, is a regulated process that eliminates damaged or unwanted cells. Inducing apoptosis in cancer cells is a common goal in cancer research and therapy. Researchers often explore different compounds or treatments that can trigger apoptosis in Hep3B cells. These may include chemotherapy drugs, targeted therapies, or experimental agents designed to promote apoptotic pathways. The cell cycle consists of different phases, and G0/G1 arrest refers to a halt in the G0 and G1 phases. This arrest prevents cells from progressing into the S phase, where DNA replication occurs.

**Figure 3:**
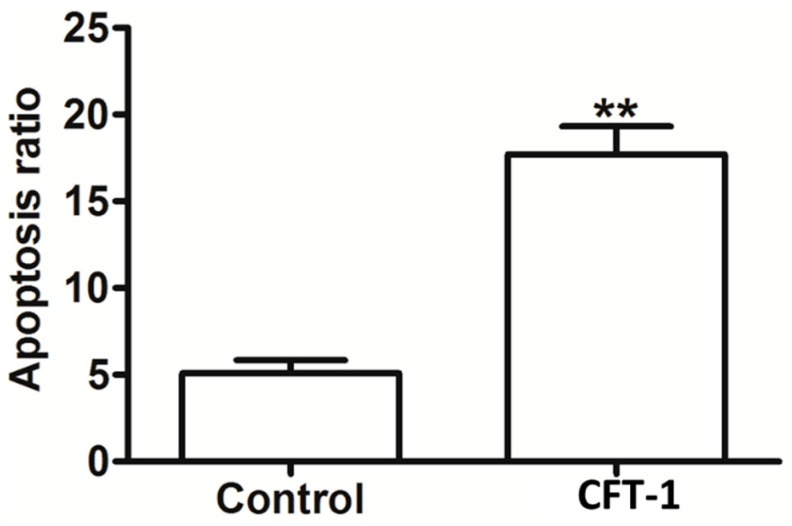
Effect of CFT-1 treatment on apoptosis of Hep3B cells. ** *p* < 0.01.

The above results confirm that CFT-1 exerts anti-cancer potential against the human Hep3B cell line by suppressing cell growth, colony formation, and cell migration. To investigate the mechanism of action, we assessed the cell death in CFT-1-treated Hep3B cells with flow cytometry. This was based on the observation of morphologic alterations caused by CFT-1, e.g., cell body shrinkage, nucleus compaction, and chromatin condensation, which together suggest an apoptotic process. We treated Hep3B cells with 143 μg/mL CFT-1 for 48 h. As shown in Figure 3, CFT-1 treatment significantly promoted apoptosis in Hep3B cells. The total apoptotic rate of Hep3B cells in the untreated control group was only 5.7%, indicating that the natural apoptotic rate of Hep3B cells was very low. Quantitative analysis indicated that the percentage of apoptotic cells increased from 5.7% to 14.8% after CFT-1 treatment.

**Figure 4:**
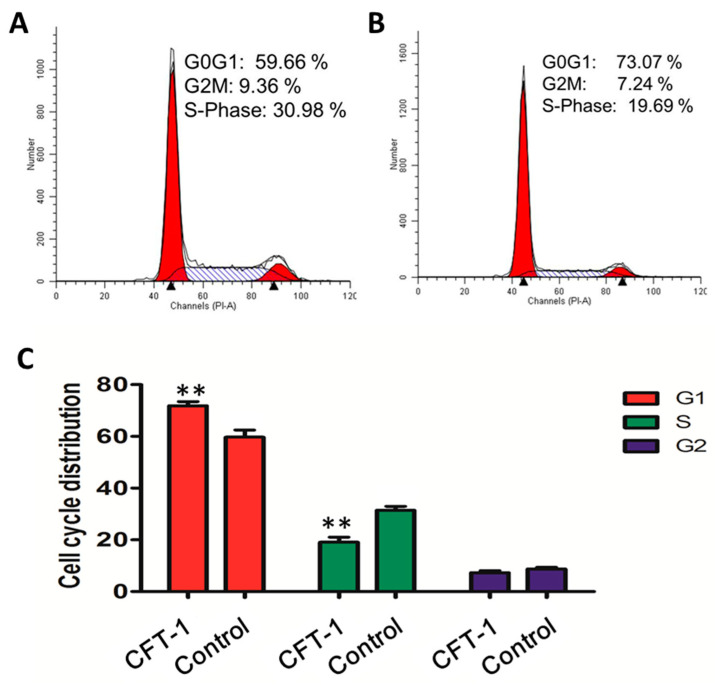
Effect of CFT-1 treatment on the Hep3B cell cycle. (**A**) The cell cycle distribution of untreated Hep3B cells. (**B**) The cell cycle distribution of Hep3B cells treated with 143 μg/mL CFT-1 for 48 h. (**C**) Quantification of cell cycle distribution results. ** *p* < 0.01.

Apoptosis and cell proliferation inhibition could be mediated by the dysregulation of cell cycle progression [28]. On this basis, we examined the cell cycle distribution with flow cytometry. After 48 h of incubation, it was shown that CFT-1 treatment resulted in a significant increase in the percentage of cells in the G0/G1 phase (73.07%) compared to the untreated cells (59.66%), while it decreased the percentage of cells in the S phase from 30.98% to 19.69% (Figure 4A–C). This is in agreement with the suppressed cell proliferation in CFT-1-treated cells. On this basis, it can be concluded that CFT-1 induces G0/G1 phase arrest in the Hep3B cell line. Notably, this conclusion coincides with previous studies on EGCG [29].

### 3.5. CFT-1 Treatment Reduces the Telomerase Activity in Hep3B Cells

Telomerase activity in liver cancer cells, such as HCC, is an area of interest in cancer research. Hep3B cell is the most common type of liver cancer, and understanding the role of telomerase in its development and progression is crucial. In many cases of liver cancer, increased telomerase activity is observed. The upregulation of telomerase allows cancer cells to maintain the lengths of their telomeres, promoting continuous cell division and contributing to the unchecked growth characteristic of cancer. Researchers study the mechanisms regulating telomerase activity in liver cancer cells to identify potential therapeutic targets. Inhibiting telomerase activity could be a strategy to limit the proliferation of liver cancer cells.

It is obvious that there is an agreement between our results for CFT-1 and previous studies on EGCG. EGCG, as the most abundant active ingredient in green tea, plays an essential role in preventing the occurrence and development of carcinomas [30,31,32]. To gain insight into the possible molecular basis behind the anti-proliferative ability of CFT-1, we referred to the available knowledge about the molecular target(s) of EGCG. According to previous studies, EGCG has been shown to inhibit telomerase activity considerably in several different cancer cell lines by repressing the target mRNA expression [33,34]. Telomerase seems to play a primary role in the cancer-fighting virtues of EGCG.

**Figure 5:**
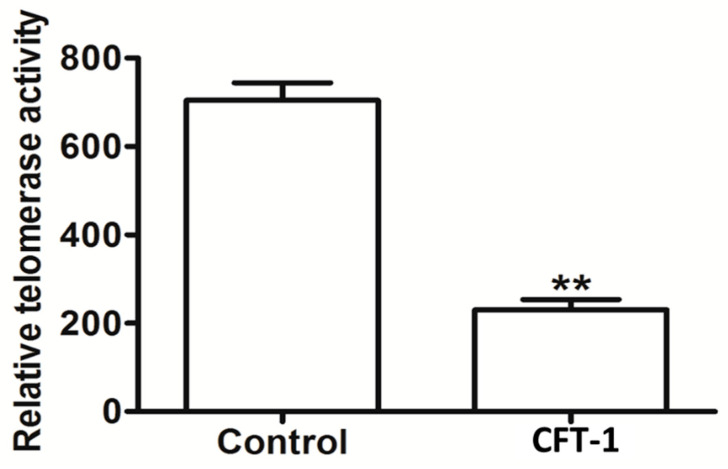
Effect of CFT-1 treatment (143 μg/mL, 48 h) on telomerase activity in Hep3B cells. ** *p* < 0.01.

On this basis, we hypothesized that CFT-1 exerts its pharmacological activity possibly through turning down the activity of telomerase. To validate this hypothesis, we examined the influence of CFT-1 extract on the telomerase activity with a telomerase–PCR–ELISA assay kit. As shown in Figure 5, CFT-1 treatment (143 μg/mL, 48 h) resulted in a significant decrease of 205.65% in the telomerase activity in Hep3B cells, suggesting a possible basis for the performance of CFT-1 on human HCC.

## 4. Discussion

Tea-based chemoprevention has gained increasing attention in recent years for the prevention of cancer. EGCG has been widely studied for its role in the chemoprevention of various tumors. Research has shown that EGCG can affect signaling pathways, induce cell apoptosis, promote cell growth arrest, and prevent cancer. As the primary active ingredient, EGCG has already shown great potential in cancer treatment. EGCG, accounting for 59% of the total catechin content, has been confirmed to have chemopreventive and chemotherapeutic effects against cancer [35]. For instance, Mayr et al. reported that EGCG also has a synergistic cytotoxic effect with conventional chemotherapy, e.g., cisplatin, in biliary tract cancer cell lines [36]. Recently, the EGCG-rich functional tea (CFT-1) has been introduced in China as a nutraceutical beverage. It is highly popular for its possible functions against cancer and other diseases. However, its health benefits and pharmacological activity remain unclear.

In the present study, we aimed to assess the anti-cancer effect of CFT-1 on the selected human Hep3B cell line. As a result, we observed dramatic alterations in cell morphology caused by CFT-1, including large amounts of cell body shrinkage, nucleus compaction, and chromatin condensation. We also found significant inhibition of proliferation, colony formation, and migration in CFT-1 treated Hep3B cells, which, together, definitely indicated the great potential of CFT-1 against human HCC. Specifically, we determined the IC50 value (143 μg/mL) of CFT-1 on Hep3B cells after 48 h of incubation. This is very close to the previously reported activity of EGCG on HCC cell lines, suggesting the strong potency of CFT-1. To gain insight into the mechanism of action, we assessed the cell death and cell cycle distribution associated with the CFT-1 treatment. Our results showed that CFT-1 significantly induced apoptosis and cell cycle arrest at the G0/G1 phase in human HCC Hep3B cells. Consistently, it was reported that EGCG inhibits cell growth, induces apoptosis, and causes G0/G1 arrest in different carcinomas [37], suggesting the similarity of action modes between CFT-1 and EGCG.

Currently available reports focus specifically on the molecular target telomerase, which is responsible for elongating telomeres [38]. Telomerase is directly implicated in tumorigenesis and is regarded as a promising anti-cancer target. To explore the possible molecular basis of the mechanism for CFT-1 tea, we assessed its influence on the telomerase activity. The CFT-1 tea has a significant inhibitory effect on tumor cell growth in vitro, and its inhibitory effect gradually increases with the increase in tea concentration. Our results showed that CFT-1 can strongly affect the enzyme in Hep3B cells by reducing the activity by more than half. On this basis, it could be suggested that CFT-1 suppresses the growth of Hep3B cells by inducing apoptosis and G0/G1 cell cycle arrest, possibly through a reduction in telomerase activity.

## 5. Conclusions

Overall, CFT-1 can inhibit the proliferation of HCC cells in vitro. Our work clearly shows the promise of CFT-1 as effective chemopreventatives and chemotherapy against human HCC. And CFT 1 tea is better than common tea in terms of protecting the liver effectively and preventing the development of cancer. These results provide a basis for the development of a functional food based on tea.

## Figures and Tables

**Table 1:** The concentrations of various compounds in CFT-1 tea extract.

Compound	Concentration (mg/g)
GA	0.39 ± 0.04
GC	1.78 ± 0.18
CAF	43.89 ± 4.4
EGC	79.73 ± 10.03
C	4.00 ± 0.29
CHL	0.14 ± 0.00
TB	0.65 ± 0.05
EC	3.25 ± 0.29
EGCG	143.83 ± 11.63
COU	0.20 ± 0.02
GCG	2.86 ± 0.22
FER	15.05 ± 1.2
SIN	0.10 ± 0.01
ECG	41.84 ± 3.20
RUT	2.07 ± 0.08
MYR	0.49 ± 0.03
QUE	0.02 ± 0.00
KAE	0.03 ± 0.00

**Table 2:** Inhibition of cell growth of Hep3B by CFT-1 extract.

Concentration of CFT-1 (μg/mL)	Growth Inhibition (%)
10	2.9 ± 0.3
30	14.6 ± 1.2
50	20.7 ± 1.1
70	28.7 ± 1.6
90	35.4 ± 2.1
100	40.3 ± 2.8
143	50.1 ± 3.1

## Data Availability

The original contributions presented in the study are included in the article/supplementary material, further inquiries can be directed to the corresponding authors.

## Supplementary Materials

The following supporting information can be downloaded at: https://www.mdpi.com/article/10.3390/foods13121867/s1, Figure S1: HPLC chromatograms of (A) CFT-1 extract and (B) standards. 1. Gallic acid (GA), 2. (−)-gallocatechin (GC), 3. caffeine (CAF), 4. theophylline (THEO), 5. (−)- epigallocatechin (EGC), 6. (+)-catechin (C), 7. chlorogenic acid (CHL), 8. theobromine (TB), 9. caffeic acid (CAA), 10. (−)-epicatechin (EC), 11. (−)-epigallocatechin gallate (EGCG), 12. ρ-coumaric acid (COU), 13. (−)-gallocatechin gallate (GCG), 14. ferulic acid (FER), 15. sinapic acid (SIN), 16. epicatechin gallate (ECG), 17. rutin (RUT), 18. myricetin (MYR), 19. quercetin (QUE), 20. kaempferol (KAE).
