# Supplementary material for: Functional Tea Extract Inhibits Cell Growth, Induces Apoptosis, and Causes G0/G1 Arrest in Human Hepatocellular Carcinoma Cell Line Possibly through Reduction in Telomerase Activity
Source: Foods. 2024 Jun 14;13(12):1867. doi: 10.3390/foods13121867 (PMC11203311; doi:10.3390/foods13121867)
Supplement: Supplementary file 1 [file foods-13-01867-s001.zip › foods-3011964-supplementary.pdf]

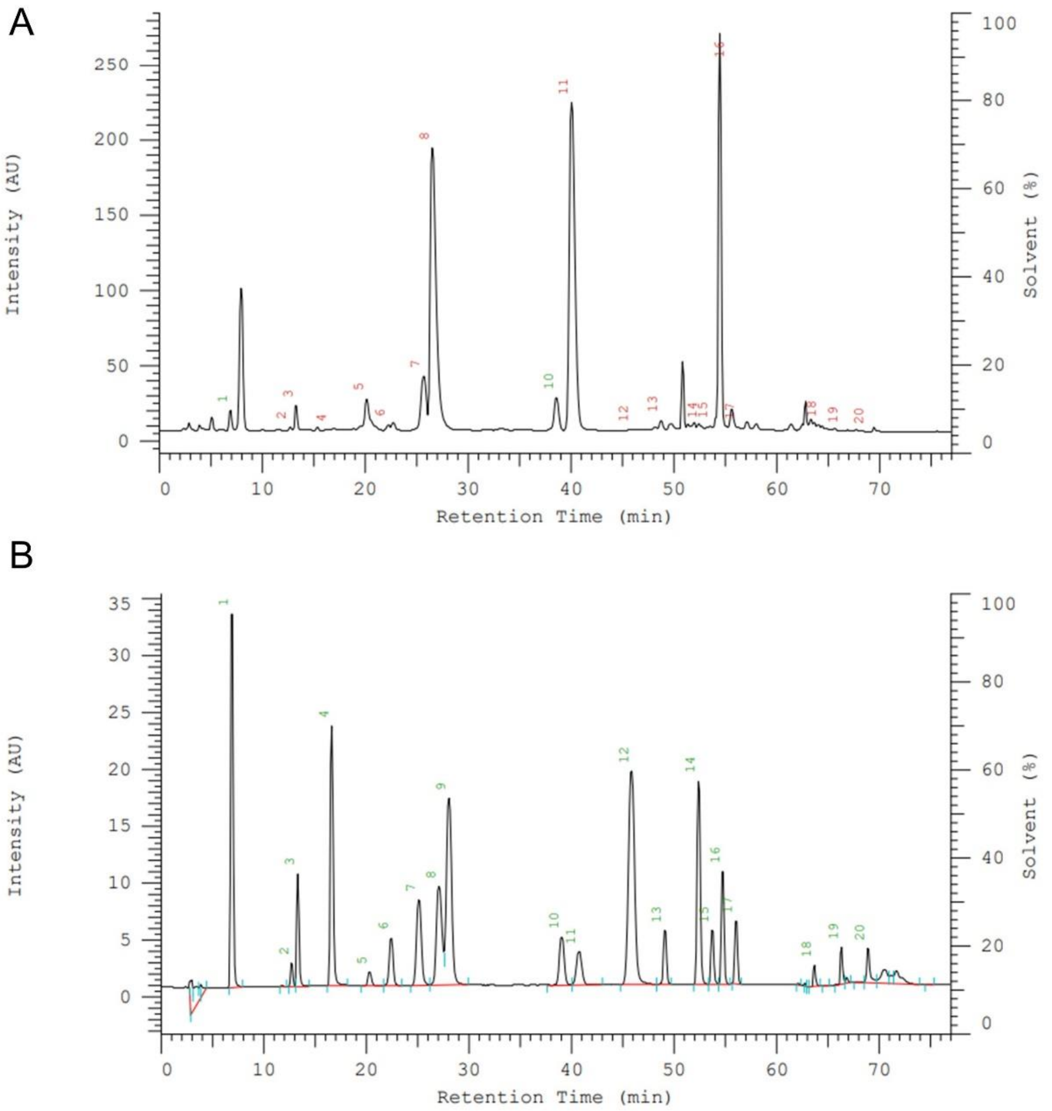

Figure S1. HPLC chromatograms of (A) CFT-1 extract and (B) standards.

1. Gallic acid (GA), 2. (−)-gallocatechin (GC), 3. caffeine (CAF), 4. theophylline (THEO), 5. (-)-epigallocatechin (EGC), 6. (+)-catechin (C), 7. chlorogenic acid (CHL), 8. theobromine (TB), 9. caffeic acid (CAA), 10. (-)-epicatechin (EC), 11. (-)-epigallocatechin gallate (EGCG), 12. ϱ-coumaric acid (COU), 13. (-)-gallocatechin gallate (GCG), 14. ferulic acid (FER), 15. sinapic acid (SIN), 16. epicatechin gallate (ECG), 17. rutin (RUT), 18. myricetin (MYR), 19. quercetin (QUE), 20. kaempferol (KAE).
